# The Sense of Quality of Life and Religious Strategies of Coping with Stress in Prison Inmates

**DOI:** 10.1007/s10943-017-0455-4

**Published:** 2017-07-26

**Authors:** Elżbieta Talik, Bartłomiej Skowroński

**Affiliations:** 1 Department of Clinical Psychology, The John Paul II Catholic University of Lublin, Al. Racławickie 14, 20-950 Lublin, Poland; 2 Institute of Social Prevention and Resocialization, Faculty of Applied Social Sciences and Resocialisation, University of Warsaw, Warsaw, Poland

**Keywords:** Religious coping, Quality of life, Offenders

## Abstract

The aim of the presented research was to analyze differences in religious strategies of coping with stress in a group of prison inmates characterized by different levels of the sense of quality of life—general, psychophysical, psychosocial, personal, and metaphysical. The participants were 390 males, aged 19–68 years, serving sentences in prisons in Poland. The measures used were the Sense of Quality of Life Questionnaire by M. Straś-Romanowska and K. I. Pargament’s RCOPE Questionnaire. As expected, individuals with a high sense of quality of life—both general and pertaining to specific dimensions—more often chose positive religious strategies, whereas participants with a low sense of quality of life more often chose negative strategies. The exception was the metaphysical aspect of the quality of life: individuals with a high intensity of this dimension more often chose some of the positive as well as negative religious strategies.

## Introduction

Imprisonment is a particularly stress-inducing situation, since it involves the frustration of numerous needs, starting from the most obvious need for freedom and autonomy, limited as a result of imprisonment (Przybilinski 2006), through deprivation of sensory stimuli, resulting from the architecture and the rules of functioning in penitentiaries, dominated by a poverty of colors as well as a monotony of space and events (Ciosek 2001). Another source of frustration is isolation from the family environment, a sense of having lost the bond with one’s family, and deprivation in the sphere of social relations, which is frequently perceived by prisoners as social rejection (Hołyst 2004). At the same time, prison inmates face the necessity to find their place in the prison hierarchy and to adapt to the norms established by the prison subculture (Chmielewska-Hampel and Wawrzyniak 2009). The temporal aspect of serving a prison sentence, connected with a sense of time being wasted, is also acutely felt by inmates (Ciosek 2001). Those and other aspects of needs deprivation experienced by inmates are not without influence on their evaluation of the quality of life understood in the most general terms as the sense of its meaningfulness, purposefulness, and agency (WHOQOL Group 1995). The quality of life comprises both objective factors, influencing an individual’s well-being, such as material situation or health, and their subjective evaluation, the sense of quality of life that relates to various aspects of human functioning: physical, psychosocial, emotional, and spiritual (Schalock 2004). In the physical dimension, well-being manifests itself in bodily health, vitality, and attractive physical appearance. Psychosocial well-being is connected with the satisfaction of the needs of belonging to a group and security and manifests itself, among other things, in establishing and maintaining bonds with other people. An extremely important aspect of the sense of quality of life is the belief in one’s own individuality and independence, associated with the possibility of making choices and bearing the responsibility for one’s own life. This personal dimension of the quality of life manifests itself in the pursuit of personal goals, interests, and passions. Finally, what is important is the possibility of realizing universal values such as good, love, truth, or beauty. The spiritual aspect of the quality of life allows a person to experience his or her own existence as going beyond earthly life (Straś-Romanowska 2005).

Research shows that imprisonment leads to a decline in the quality of life (Coid 1993; Williams 2003), particularly as regards its psychosocial aspect (Dolińska-Zygmunt and Mokrzyńska 2013). A symptom of the lowered sense of quality of life is an increase in anxiety and depression (Chmielewska-Hampel and Wawrzyniak 2009) and a decline in emotional intelligence (Dolińska-Zygmunt and Mokrzyńska 2013). The sense of quality of life in prison inmates correlates positively with optimism and with future time perspective (Dolińska-Zygmunt and Mokrzyńska 2013). An interesting comparative study was carried out by Bouman and colleagues (2008), who assessed differences in the sense of quality of life in two groups of offenders: convicted for sex crimes and for other kinds of violence. The analyses revealed that sex offenders had a higher general sense of quality of life as well as a higher sense of quality of life regarding health, security, and relations with the family compared to nonsex offenders. Other research reveals that this group of convicted offenders—additionally, with symptoms of intellectual disability—was characterized by a lower sense of quality of life regarding relations with others and the experience of entertainment compared to inmates not guilty of sex crimes (Stepstoe et al. 2006).

Imprisonment—by lowering the sense of quality of life in prison inmates—is a particularly stress-inducing situation (Chmielewska-Hampel and Wawrzyniak 2009; Niewiadomska 2011), which inmates try to cope with using various remedial strategies. Research so far has taken into account mainly the traditional ways of coping with stress (cf. Shulman and Cauffman 2011; Connor-Smith et al. 2000; Wadsworth and Compas 2002; Looman et al. 2004; Cortoni et al. 1999), overlooking religious strategies, whose essence lies in reference to religion—or, more accurately, to the sphere of the sacred (Pargament 1997). Some religious strategies have a positive character, for example, seeking a way to change one’s life in religion, and these usually involve better adaptation—they reduce the indicators of depression as well as correlate with higher self-esteem, satisfaction with life, and quality of life (Harrison et al. 2001). By contrast, negative religious strategies, such as perceiving God as punishing for sins, are usually connected with worse adaptation and various forms of psychopathology, including anxieties, phobias, depressions, obsessive–compulsive disorders, and somatizations (Ano and Vasconcelles 2005; Pearce et al. 2006).

No research has been found concerning the relations between the sense of quality of life and religious coping with stress in prison inmates. The available publications relate to other social groups—usually ill people (Lee et al. 2014; Pedersen et al. 2013; Ramirez et al. 2012; Tedrus et al. 2013; Warren et al. 2015), including psychiatric patients (Nolan et al. 2012), as well as their caregivers (Pearce et al. 2006), paramedics (Prati et al. 2011), and immigrants (Dunn and O’Brien 2009). In the studies mentioned, the analyses concerned the influence of religious strategies on the quality of life—their findings consistently showed that negative religious strategies lower the quality of life (Pedersen et al. 2013; Tedrus et al. 2013), as opposed to positive strategies, associated with a higher sense of quality of life (Nolan et al. 2012; Ramirez et al. 2012; Warren et al. 2015). The reverse relationship—the choice of religious strategies by people with different levels of the sense of quality of life—was not analyzed.

Several studies have been carried out in a group of inmates concerning religious coping with stress in the context of variables other than the sense of quality of life. Analyses performed by Pallas (2014) reveal that in males convicted for sex crimes negative religious strategies correlate positively with anxiety and depression, as opposed to positive strategies, which reduce the level of anxiety. Similar results were obtained in the study by Allen and colleagues (2013)—positive religious strategies reduced the level of depression, while negative ones not only increased the level of depression but were also significantly related to a desire to die soon in elderly prisoners serving a sentence for murder. In a Polish study by Niewiadomska (2011), both types of religious strategies turned out not to be significant to the following aspects of prisoners’ sense of social rootedness: their sense of meaning, resourcefulness, and autonomy and their perception of social order.

The little number of studies on religious coping with stress in prison inmates (only one study is available in Poland—the one by Niewiadomska 2011) and the lack of analyses concerning the relationship between the sense of quality of life and religious strategies justified taking up the present research, whose aim was to analyze differences in religious strategies of coping with stress in a group of prison inmates characterized by different levels of the sense of quality of life.

## Method

The aim of the study was to investigate the differences in religious strategies of coping with stress between groups of inmates distinguished according to their sense of quality of life. With the aim thus defined, the following research problem was formulated: what are the differences in religious strategies of coping with stress between groups of inmates characterized by different levels of the sense of quality of life—general and pertaining to particular dimensions?

The following research hypotheses were formulated:

### **H1**

Individuals with a high general sense of quality of life statistical significantly more often use positive religious strategies than individuals with a low general sense of quality of life.

### **H2**

Individuals with a low psychophysical sense of quality of life statistical significantly more often use negative religious strategies than individuals with a high psychophysical sense of quality of life.

### **H3**

Individuals with a high psychosocial sense of quality of life statistical significantly more often use positive religious strategies than individuals with a low psychosocial sense of quality of life.

### **H4**

Individuals with a low personal sense of quality of life statistical significantly more often use negative religious strategies than individuals with a high personal sense of quality of life.

### **H5**

Individuals with a high metaphysical sense of quality of life statistical significantly more often use positive religious strategies than individuals with a low metaphysical sense of quality of life.

In order to verify the hypotheses, research was conducted on a group of 390 male prison inmates aged 19–68 years (*M* = 35.19, SD = 9.65). The largest number of people had vocational (26.7%) and elementary education (18.5%); only 7.7% of the sample were people with higher education. About forty percent (38.2%) of the inmates came from big cities (above 150 thousand inhabitants). The group of male prison inmates was chosen on the basis of convenience sampling and was selected from penitentiary facilities administrated by the District Inspectorate of Prison Service in Warsaw, and in particular: at the Warsaw-Grochów, Warsaw-Białołęka, Warsaw-Mokotów, and Warsaw-Służewiec Remand Prisons as well as at the Warsaw-Białołęka Penitentiary. The study was conducted in April 2014.

A strong majority of the participants declared Roman Catholicism (76.4%), and 12.3%—no religion at all; 3.8% of the sample were Protestant and 3.6% were Orthodox. About 68% were believers and strong believers; weak believers constituted 17.9% of the sample and nonbelievers—14.9%. Most participants (71.3%) engaged in religious practices.

About sixty percent (59.2%) of the inmates had started serving their sentences in the years 2000–2014; 17.4% of the inmates will complete serving it in 2015, and 14.1%—in the years 2018–2050.

The following instruments were used: (1) The Sense of Quality of Life Questionnaire and (2) RCOPE Questionnaire.

*The Sense of Quality of Life Questionnaire (SQLQ)* by Maria Straś Romanowska measures the general sense of quality of life and its four dimensions: psychophysical, psychosocial, personal, and metaphysical (Straś-Romanowska and Frąckowiak 2007). The instrument consists of 60 statements. The reliability of the scale—its test–retest stability (over a 3-week interval) is *r* = .81 in a group of young people, *r* = .73 in a group of elderly people, and *r* = .65 in adults. The internal consistency of the scale, assessed using the Cronbach’s alpha coefficient, is the following, respectively: *α* = .77 for the Psychophysical Sense of Quality of Life scale; *α* = .71 for the Psychosocial scale; *α* = .72 for the Personal scale; *α* = .65 for the Metaphysical scale; and *α* = .70 for the whole SQLQ. The construct validity of the SQLQ was assessed, e.g., based on the correlations of particular scales with other measures; the correlations obtained were statistically significant and ranged from .30 (Psychophysical Sense of Quality of Life scale) to .53 (Personal Sense of Quality of Life scale).

*RCOPE Questionnaire* by Kenneth I. Pargament measures religious strategies of coping with stress. It consists of 105 items, grouped into 17 scales measuring religious strategies: positive (10 scales—e.g., *Benevolent Religious Reappraisal/Spiritual Support; Collaborative Religious Coping; Active Religious Surrender*) and negative (7 scales—e.g., *Punishing God Reappraisal*; *Demonic Reappraisal*; *Reappraisal of God’s Power*; *Passive Religious Deferral*). The participants’ task was to rate the extent to which they used each way of religious coping with negative events by choosing one of the responses on a four-point Likert scale (from 0—*not at all*, do 3—*to a large extent*). The reliability—internal consistency—of the questionnaire ranges from .61 (*Marking Religious Boundaries*) to .94 (*Religious Direction/Conversion*). The validity of the RCOPE Questionnaire was assessed, e.g., through confirmatory factor analysis performed in a group of elderly hospitalized people (*N* = 551) (cf. Pargament, Koenig, and Perez 2000). A Polish translation of the measure (cf. Talik and Szewczyk 2008) was used in the present study, with the original scales employed.

## Results

The analysis of empirical data started with computing the descriptive statistics for each variable—the sense of quality of life and its specific dimensions (Table 1) as well as positive and negative religious strategies (Table 2).

**Table 1:** Descriptive statistics for the sense of quality of life and its dimensions (*N* = 390)

	Min	Max	*M*	SD
Psychophysical sense of quality of life	1.40	4.00	3.06	.49
Psychosocial sense of quality of life	1.53	3.87	2.84	.41
Personal sense of quality of life	1.40	4.00	3.02	.42
Metaphysical sense of quality of life	1.40	4.00	3.03	.45
General sense of quality of life	1.55	3.85	2.99	.37

**Table 2:** Descriptive statistics for positive and negative religious strategies (*N* = 390)

Positive (+) and Negative (−) religious strategies	Min	Max	*M*	SD
Benevolent religious reappraisal/spiritual support (+)	0	3	1.21	.85
Punishing god reappraisal (−)	0	3	1.09	.78
Demonic reappraisal (−)	0	3	1.02	.89
Reappraisal of god’s power (−)	0	3	1.13	.77
Collaborative religious coping (+)	0	3	1.38	.55
Active religious surrender (+)	0	3	1.11	.83
Passive religious deferral (−)	0	3	.82	.83
Pleading for direct intercession (−)	0	3	1.18	.77
Religious focus (+)	0	3	1.00	.80
Religious purification/forgiving (+)	0	3	1.31	.87
Spiritual connection (+)	0	3	1.20	.95
Spiritual discontent (−)	0	3	1.08	.74
Marking religious boundaries (+)	0	3	1.24	.83
Seeking support from clergy/members (+)	0	3	1.02	.88
Religious helping (+)	0	3	1.08	.81
Interpersonal religious discontent (−)	0	3	1.04	.75
Religious direction/conversion (+)	0	3	1.19	.89
Positive religious strategies (+)	0	2.91	1.19	.71
Negative religious strategies (−)	0	3	1.05	.67

Prison inmates’ ratings were the highest in the case of the psychophysical dimension of the sense of quality of life (*M* = 3.06, SD = .49); they were similar in the case of metaphysical (*M* = 3.03, SD = .45) and personal (*M* = 3.02, SD = .42) sense of quality of life. The lowest-rated dimension was the psychosocial sense of quality of life (*M* = 2.84, SD = .41).

Of the positive religious strategies, the participants scored highest on the *Collaborative Religious Coping* scale (*M* = 1.38, SD = .55) and the lowest on the *Religious Focus* scale (*M* = 1.00, SD = .80). The highest-rated negative religious strategy was *Pleading for Direct Intercession* (*M* = 1.18, SD = .77) and the lowest-rated one was *Passive Religious Deferral* (*M* = .82, SD = .83). The scores were higher for positive religious strategies (*M* = 1.19, SD = .71) than for negative ones (*M* = 1.05, SD = .67).

In order to test the hypotheses advanced, analysis of variance (ANOVA) was performed. A Hochberg G2 post hoc test was used—due to the homogeneity of the variance in all the variables and the unequal size of the groups. Three groups of prison inmates were distinguished, with different levels of the general sense of quality of life and its specific dimensions. The first group was individuals with a high sense of quality of life, the second group was those with its medium level, and the third group—with a low sense of quality of life. The groups were distinguished on the basis of the sten norms developed by Straś-Romanowska (2005).^1^

### General Sense of Quality of Life and Religious Strategies of Coping with Stress

There were statistically significant differences between people with a high (Group 3) versus low (Group 1) general sense of quality of life in some of the positive and negative religious strategies (Table 3).

**Table 3:** Differences in religious strategies between individuals with different levels of the general sense of quality of life: low (1), medium (2), and high (3)

Positive (+) and Negative (−) religious strategies	Levels of the general sense of quality of life	*N*	*M*	SD	*F*	*p*	Post hoc
Benevolent religious reappraisal/spiritual support (+)	Low (1)	151	1.14	.78	4.03	.019	1 < 3 2 < 3
	Medium (2)	194	1.18	.87			
	High (3)	45	1.54	.90			
	Total	390	1.21	.85			
Punishing god reappraisal (−)	Low (1)	151	1.12	.76	.26	.775	ns
	Medium (2)	194	1.07	.80			
	High (3)	45	1.12	.76			
	Total	390	1.09	.78			
Demonic reappraisal (−)	Low (1)	151	1.10	.83	1.17	.311	ns
	Medium (2)	194	.96	.92			
	High (3)	45	1.02	.96			
	Total	390	1.02	.89			
Reappraisal of god’s power (−)	Low (1)	151	1.24	.80	2.63	.074	ns
	Medium (2)	194	1.07	.76			
	High (3)	45	1.02	.73			
	Total	390	1.13	.77			
Collaborative religious coping (+)	Low (1)	151	1.33	.47	13.54	.000	1 < 3 2 < 3
	Medium (2)	194	1.33	.57			
	High (3)	45	1.77	.59			
	Total	390	1.38	.56			
Active religious surrender (+)	Low (1)	151	1.09	.80	1.76	.173	ns
	Medium (2)	194	1.08	.85			
	High (3)	45	1.33	.83			
	Total	390	1.11	.83			
Passive religious deferral (−)	Low (1)	151	1.05	.82	9.16	.000	1 > 2 1 > 3
	Medium (2)	194	.69	.80			
	High (3)	45	.68	.83			
	Total	390	.82	.83			
Pleading for direct intercession (−)	Low (1)	151	1.20	.76	1.25	.288	ns
	Medium (2)	194	1.13	.79			
	High (3)	45	1.32	.76			
	Total	390	1.18	.77			
Religious focus (+)	Low (1)	151	1.06	.82	2.48	.085	ns
	Medium (2)	194	.91	.75			
	High (3)	45	1.15	.90			
	Total	390	.99	.80			
Religious purification/forgiving (+)	Low (1)	151	1.19	.81	6.82	.001	1 < 3 2 < 3
	Medium (2)	194	1.30	.89			
	High (3)	45	1.73	.91			
	Total	390	1.31	.87			
Spiritual connection (+)	Low (1)	151	1.12	.89	5.38	.005	1 < 3 2 < 3
	Medium (2)	194	1.16	.96			
	High (3)	45	1.63	1.00			
	Total	390	1.20	.95			
Spiritual discontent (−)	Low (1)	151	1.20	.72	3.50	.031	ns
	Medium (2)	194	1.02	.74			
	High (3)	45	.94	.77			
	Total	390	1.08	.74			
Marking religious boundaries (+)	Low (1)	151	1.24	.84	.35	.703	ns
	Medium (2)	194	1.22	.81			
	High (3)	45	1.33	.89			
	Total	390	1.24	.83			
Seeking support from clergy/members (+)	Low (1)	151	1.07	.88	2.54	.080	ns
	Medium (2)	194	.94	.87			
	High (3)	45	1.24	.91			
	Total	390	1.02	.88			
Religious helping (+)	Low (1)	151	1.02	.79	4.25	.015	1 < 3 2 < 3
	Medium (2)	194	1.05	.80			
	High (3)	45	1.41	.87			
	Total	390	1.08	.81			
Interpersonal religious discontent (−)	Low (1)	151	1.16	.78	3.48	.032	1 > 2
	Medium (2)	194	.95	.72			
	High (3)	45	1.05	.73			
	Total	390	1.04	.75			
Religious direction/conversion (+)	Low (1)	151	1.13	.81	3.15	.044	1 < 3
	Medium (2)	194	1.16	.92			
	High (3)	45	1.50	.95			
	Total	390	1.19	.89			
Positive religious strategies (+)	Low (1)	151	1.15	.68	4.81	.009	1 < 3 2 < 3
	Medium (2)	194	1.15	.72			
	High (3)	45	1.50	.76			
	Total	390	1.19	.72			
Negative religious strategies (−)	Low (1)	151	1.15	.68	2.87	.058	ns
	Medium (2)	194	.98	.66			
	High (3)	45	1.02	.62			
	Total	390	1.05	.67			

Individuals with a high general sense of quality of life (Group 3) chose some of the positive religious strategies significantly more often than individuals with a low level of this variable (Group 1), namely: *Benevolent Religious Reappraisal/Spiritual Support* (*F* = 4.03, *p* < .05), *Collaborative Religious Coping* (*F* = 13.53, *p* < .001), *Religious Purification/Forgiveness* (*F* = 6.82, *p* < .001), *Spiritual Connection* (*F* = 5.38, *p* < .01), *Religious Helping* (*F* = 4.25, *p* < .05), *Religious Direction/Conversion* (*F* = 3.15, *p* < .05), and *Positive Religious Coping* treated as a whole (*F* = 4.81, *p* < .01)—the first hypothesis was confirmed. Individuals with a low general sense of quality of life chose the strategy of *Passive Religious Deferral* (*F* = 9.16; *p* < .001) significantly more often than participants with a high level of this variable (Group 3).

### Psychophysical Sense of Quality of Life and Religious Strategies of Coping with Stress

There were statistically significant differences between people with a high (Group 3) versus low (Group 1) psychophysical sense of quality of life in some of the positive and negative religious strategies (Table 4).

Individuals with a low psychophysical sense of quality of life (Group 1) chose some of the negative religious strategies significantly more often than individuals with a high level of this variable (Group 3), namely: *Punishing God Reappraisal* (*F* = 3.62, *p* < .05*), Demonic Reappraisal* (*F* = 3.84, *p* < .05), *Reappraisal of God’s Power* (*F* = 5.48, *p* < .01), *Passive Religious Deferral* (*F* = 9.66, *p* < .001), *Pleading for Direct Intercession* (*F* = 3.39, *p* < .05), *Spiritual Discontent* (*F* = 6.64, *p* < .001), and *Interpersonal Religious Discontent* (*F* = 7.37, *p* < .001) as well as score higher on *Negative Religious Coping* treated as a whole (*F* = 7.40, *p* < .001). Hypothesis 2 was confirmed (Table 4).

**Table 4:** Differences in religious strategies between individuals with different levels of the psychophysical sense of quality of life: low (1), medium (2), and high (3)

Positive (+) and Negative (−) religious strategies	Levels of the psychophysical sense of quality of life	*N*	*M*	SD	*F*	*p*	Post hoc
Benevolent religious reappraisal/spiritual support (+)	Low (1)	82	1.18	.72	2.06	.129	ns
	Medium (2)	250	1.26	.87			
	High (3)	58	1.02	.91			
	Total	390	1.21	.85			
Punishing god reappraisal (−)	Low (1)	82	1.19	.68	3.62	.028	1 > 3 2 > 3
	Medium (2)	250	1.12	.80			
	High (3)	58	.85	.81			
	Total	390	1.10	.78			
Demonic reappraisal (−)	Low (1)	82	1.16	.80	3.84	.022	1 > 3
	Medium (2)	250	1.04	.89			
	High (3)	58	.74	.97			
	Total	390	1.02	.89			
Reappraisal of god’s power (−)	Low (1)	82	1.26	.77	5.48	.005	1 > 3 2 > 3
	Medium (2)	250	1.15	.77			
	High (3)	58	.84	.73			
	Total	390	1.13	.77			
Collaborative religious coping (+)	Low (1)	82	1.37	.46	.06	.944	ns
	Medium (2)	250	1.39	.55			
	High (3)	58	1.36	.71			
	Total	390	1.38	.56			
Active religious surrender (+)	Low (1)	82	1.14	.73	2.26	.106	ns
	Medium (2)	250	1.15	.86			
	High (3)	58	.90	.81			
	Total	390	1.11	.83			
Passive religious deferral (−)	Low (1)	82	1.10	.78	9.66	.000	1 > 2 1 > 3 2 > 3
	Medium (2)	250	.81	.83			
	High (3)	58	.49	.74			
	Total	390	.83	.83			
Pleading for direct intercession (−)	Low (1)	82	1.23	.73	3.39	.035	1 > 3 2 > 3
	Medium (2)	250	1.22	.78			
	High (3)	58	.94	.79			
	Total	390	1.18	.77			
Religious focus (+)	Low (1)	82	1.08	.73	2.90	.056	ns
	Medium (2)	250	1.03	.81			
	High (3)	58	.77	.82			
	Total	390	1.00	.80			
Religious purification/forgiving (+)	Low (1)	82	1.26	.78	.47	.627	ns
	Medium (2)	250	1.34	.88			
	High (3)	58	1.23	.97			
	Total	390	1.31	.87			
Spiritual connection (+)	Low (1)	82	1.17	.80	.92	.400	ns
	Medium (2)	250	1.24	.96			
	High (3)	58	1.06	1.09			
	Total	390	1.20	.95			
Spiritual discontent (−)	Low (1)	82	1.28	.67	6.64	.001	1 > 3 2 > 3
	Medium (2)	250	1.08	.76			
	High (3)	58	.82	.68			
	Total	390	1.08	.74			
Marking religious boundaries (+)	Low (1)	82	1.24	.77	2.92	.055	ns
	Medium (2)	250	1.30	.85			
	High (3)	58	1.00	.82			
	Total	390	1.24	.83			
Seeking support from clergy/members (+)	Low (1)	82	1.13	.84	1.91	.149	ns
	Medium (2)	250	1.03	.90			
	High (3)	58	.83	.83			
	Total	390	1.02	.88			
Religious helping (+)	Low (1)	82	1.08	.74	1.71	.182	ns
	Medium (2)	250	1.12	.83			
	High (3)	58	.90	.84			
	Total	390	1.08	.81			
Interpersonal religious discontent (−)	Low (1)	82	1.25	.73	7.37	.001	1 > 3 2 > 3
	Medium (2)	250	1.04	.76			
	High (3)	58	.77	.64			
	Total	390	1.04	.75			
Religious direction/conversion (+)	Low (1)	82	1.21	.75	1.48	.229	ns
	Medium (2)	250	1.22	.90			
	High (3)	58	1.00	1.01			
	Total	390	1.19	.89			
Positive religious strategies (+)	Low (1)	82	1.20	.62	1.63	.197	ns
	Medium (2)	250	1.23	.72			
	High (3)	58	1.04	.80			
	Total	390	1.19	.72			
Negative religious strategies (−)	Low (1)	82	1.21	.64	7.40	.001	1 > 3 2 > 3
	Medium (2)	250	1.06	.67			
	High (3)	58	.78	.64			
	Total	390	1.05	.67			

### Psychosocial Sense of Quality of Life and Religious Strategies of Coping with Stress

There were statistically significant differences between people with a high (Group 3) versus low (Group 1) psychosocial sense of quality of life in some of the positive religious strategies (Table 5).

**Table 5:** Differences in religious strategies between individuals with different levels of the psychosocial sense of quality of life: low (1), medium (2), and high (3)

Positive (+) and Negative (−) religious strategies	Levels of the psychosocial sense of quality of life	*N*	*M*	SD	*F*	*p*	Post hoc
Benevolent religious reappraisal/spiritual support (+)	Low (1)	136	1.16	.76	.92	.401	ns
	Medium (2)	215	1.21	.89			
	High (3)	39	1.37	.88			
	Total	390	1.21	.85			
Punishing god reappraisal (−)	Low (1)	136	1.16	.76	.72	.487	ns
	Medium (2)	215	1.05	.80			
	High (3)	39	1.10	.70			
	Total	390	1.10	.78			
Demonic reappraisal (−)	Low (1)	136	1.13	.86	1.50	.224	ns
	Medium (2)	215	.96	.90			
	High (3)	39	1.00	.95			
	Total	390	1.02	.89			
Reappraisal of god’s power (−)	Low (1)	136	1.23	.74	1.72	.180	ns
	Medium (2)	215	1.08	.80			
	High (3)	39	1.06	.75			
	Total	390	1.13	.77			
Collaborative religious coping (+)	Low (1)	136	1.27	.47	4.97	.007	1 < 3
	Medium (2)	215	1.41	.56			
	High (3)	39	1.56	.75			
	Total	390	1.38	.56			
Active religious surrender (+)	Low (1)	136	1.08	.76	1.19	.307	ns
	Medium (2)	215	1.10	.87			
	High (3)	39	1.30	.81			
	Total	390	1.11	.83			
Passive religious deferral (−)	Low (1)	136	1.01	.82	5.13	.006	1 > 2
	Medium (2)	215	.74	.81			
	High (3)	39	.69	.84			
	Total	390	.83	.83			
Pleading for direct intercession (−)	Low (1)	136	1.19	.73	.03	.972	ns
	Medium (2)	215	1.17	.80			
	High (3)	39	1.17	.81			
	Total	390	1.18	.77			
Religious focus (+)	Low (1)	136	1.01	.79	.47	.627	ns
	Medium (2)	215	.97	.78			
	High (3)	39	1.10	.94			
	Total	390	1.00	.80			
Religious purification/forgiving (+)	Low (1)	136	1.22	.80	3.77	.024	1 < 3 2 < 3
	Medium (2)	215	1.30	.89			
	High (3)	39	1.65	.98			
	Total	390	1.31	.87			
Spiritual connection (+)	Low (1)	136	1.14	.88	2.56	.078	ns
	Medium (2)	215	1.18	.97			
	High (3)	39	1.52	1.04			
	Total	390	1.20	.95			
Spiritual discontent (−)	Low (1)	136	1.20	.70	2.82	.061	ns
	Medium (2)	215	1.02	.75			
	High (3)	39	1.02	.82			
	Total	390	1.08	.74			
Marking religious boundaries (+)	Low (1)	136	1.16	.76	1.08	.339	ns
	Medium (2)	215	1.28	.86			
	High (3)	39	1.33	.93			
	Total	390	1.24	.83			
Seeking support from clergy/members (+)	Low (1)	136	.98	.82	2.47	.086	ns
	Medium (2)	215	1.00	.89			
	High (3)	39	1.32	1.00			
	Total	390	1.02	.88			
Religious helping (+)	Low (1)	136	1.06	.77	2.74	.066	ns
	Medium (2)	215	1.04	.81			
	High (3)	39	1.37	.93			
	Total	390	1.08	.81			
Interpersonal religious discontent (−)	Low (1)	136	1.17	.76	3.13	.045	1 > 2
	Medium (2)	215	.96	.73			
	High (3)	39	1.06	.77			
	Total	390	1.04	.75			
Religious direction/conversion (+)	Low (1)	136	1.18	.81	1.21	.300	ns
	Medium (2)	215	1.16	.91			
	High (3)	39	1.39	1.01			
	Total	390	1.19	.89			
Positive religious strategies (+)	Low (1)	136	1.14	.64	2.22	.110	ns
	Medium (2)	215	1.18	.74			
	High (3)	39	1.42	.82			
	Total	390	1.19	.72			
Negative religious strategies (−)	Low (1)	136	1.15	.64	2.41	.091	ns
	Medium (2)	215	1.00	.69			
	High (3)	39	1.01	.62			
	Total	390	1.05	.67			

Individuals with a high psychosocial sense of quality of life (Group 3) chose some of the positive religious strategies significantly more often than individuals with a low level of this variable (Group 1), namely: *Collaborative Religious Coping* (*F* = 4.97, *p* < .01) and *Religious Purification/Forgiveness* (*F* = 3.77, *p* < .05). Hypothesis 3 was confirmed.

### Personal Sense of Quality of Life and Religious Strategies of Coping with Stress

There were statistically significant differences between people with a high (Group 3) versus low (Group 1) personal sense of quality of life in one negative religious strategy (Table 6).

**Table 6:** Differences in religious strategies between individuals with different levels of the personal sense of quality of life: low (1), medium (2), and high (3)

Positive (+) and Negative (−) religious strategies	Levels of the personal sense of quality of life	*N*	*M*	SD	*F*	*p*	Post hoc
Benevolent religious reappraisal/spiritual support (+)	Low (1)	83	1.09	.76	1.22	.296	ns
	Medium (2)	264	1.22	.85			
	High (3)	43	1.33	.96			
	Total	390	1.21	.85			
Punishing god reappraisal (−)	Low (1)	83	1.06	.74	.50	.607	ns
	Medium (2)	264	1.12	.78			
	High (3)	43	1.00	.86			
	Total	390	1.10	.78			
Demonic reappraisal (−)	Low (1)	83	1.11	.81	2.31	.101	ns
	Medium (2)	264	1.04	.92			
	High (3)	43	.76	.86			
	Total	390	1.02	.89			
Reappraisal of god’s power (−)	Low (1)	83	1.17	.78	1.19	.305	ns
	Medium (2)	264	1.14	.78			
	High (3)	43	.96	.74			
	Total	390	1.13	.77			
Collaborative religious coping (+)	Low (1)	83	1.35	.45	2.21	.111	ns
	Medium (2)	264	1.36	.54			
	High (3)	43	1.55	.78			
	Total	390	1.38	.56			
Active religious surrender (+)	Low (1)	83	1.05	.81	.42	.660	ns
	Medium (2)	264	1.12	.81			
	High (3)	43	1.18	.96			
	Total	390	1.11	.83			
Passive religious deferral (−)	Low (1)	83	1.01	.83	4.22	.015	1 > 3
	Medium (2)	264	.81	.82			
	High (3)	43	.57	.83			
	Total	390	.83	.83			
Pleading for direct intercession (−)	Low (1)	83	1.19	.76	.01	.995	ns
	Medium (2)	264	1.18	.78			
	High (3)	43	1.19	.81			
	Total	390	1.18	.77			
Religious focus (+)	Low (1)	83	.99	.80	.16	.856	ns
	Medium (2)	264	1.01	.78			
	High (3)	43	.94	.90			
	Total	390	1.00	.80			
Religious purification/forgiving (+)	Low (1)	83	1.16	.83	2.22	.110	ns
	Medium (2)	264	1.32	.86			
	High (3)	43	1.50	1.03			
	Total	390	1.31	.87			
Spiritual connection (+)	Low (1)	83	1.08	.89	.88	.416	ns
	Medium (2)	264	1.23	.96			
	High (3)	43	1.27	1.02			
	Total	390	1.20	.95			
Spiritual discontent (−)	Low (1)	83	1.15	.73	1.92	.148	ns
	Medium (2)	264	1.10	.73			
	High (3)	43	.88	.82			
	Total	390	1.08	.74			
Marking religious boundaries (+)	Low (1)	83	1.26	.83	.03	.969	ns
	Medium (2)	264	1.24	.84			
	High (3)	43	1.23	.80			
	Total	390	1.24	.83			
Seeking support from clergy/members (+)	Low (1)	83	1.00	.82	.10	.905	ns
	Medium (2)	264	1.04	.91			
	High (3)	43	.99	.86			
	Total	390	1.02	.88			
Religious helping (+)	Low (1)	83	.95	.75	1.34	.263	ns
	Medium (2)	264	1.11	.82			
	High (3)	43	1.16	.88			
	Total	390	1.08	.81			
Interpersonal religious discontent (−)	Low (1)	83	1.16	.80	1.48	.230	ns
	Medium (2)	264	1.02	.74			
	High (3)	43	.94	.70			
	Total	390	1.04	.75			
Religious direction/conversion (+)	Low (1)	83	1.08	.81	.97	.382	ns
	Medium (2)	264	1.20	.90			
	High (3)	43	1.29	.98			
	Total	390	1.19	.89			
Positive religious strategies (+)	Low (1)	83	1.11	.66	.92	.400	ns
	Medium (2)	264	1.20	.71			
	High (3)	43	1.28	.83			
	Total	390	1.19	.72			
Negative religious strategies (−)	Low (1)	83	1.12	.67	1.56	.212	ns
	Medium (2)	264	1.06	.67			
	High (3)	43	.90	.64			
	Total	390	1.05	.67			

Hypothesis 4 was thus confirmed for one negative religious strategy: *Passive Religious Deferral* (*F* = 4.22, *p* < .05), on which higher scores were obtained by individuals with a low personal sense of quality of life.

### Metaphysical Sense of Quality of Life and Religious Strategies of Coping with Stress

There were statistically significant differences between people with a high (Group 3) versus low (Group 1) metaphysical sense of quality of life in some of the positive and negative religious strategies (Table 7).

**Table 7:** Differences in religious strategies between individuals with different levels of the metaphysical sense of quality of life: low (1), medium (2), and high (3)

Positive (+) and Negative (−) religious strategies	Levels of the metaphysical sense of quality of life	*N*	*M*	SD	*F*	*p*	Post hoc
Benevolent religious reappraisal/spiritual support (+)	Low (1)	148	.95	.77	20.82	.000	1 < 2 1 < 3 2 < 3
	Medium (2)	226	1.31	.83			
	High (3)	16	2.17	.84			
	Total	390	1.21	.85			
Punishing god reappraisal (−)	Low (1)	148	.93	.75	10.66	.000	1 < 2 1 < 3 2 < 3
	Medium (2)	226	1.15	.77			
	High (3)	16	1.79	.70			
	Total	390	1.10	.78			
Demonic reappraisal (−)	Low (1)	148	.94	.80	8.50	.000	1 < 3 2 < 3
	Medium (2)	226	1.01	.91			
	High (3)	16	1.89	1.04			
	Total	390	1.02	.89			
Reappraisal of god’s power (−)	Low (1)	148	1.11	.82	.17	.844	ns
	Medium (2)	226	1.14	.75			
	High (3)	16	1.22	.76			
	Total	390	1.13	.77			
Collaborative religious coping (+)	Low (1)	148	1.28	.52	11.02	.000	1 < 3 2 < 3
	Medium (2)	226	1.41	.56			
	High (3)	16	1.93	.53			
	Total	390	1.38	.56			
Active religious surrender (+)	Low (1)	148	.91	.79	9.49	.000	1 < 2 1 < 3
	Medium (2)	226	1.21	.82			
	High (3)	16	1.61	.80			
	Total	390	1.11	.83			
Passive religious deferral (−)	Low (1)	148	.89	.81	1.42	.243	ns
	Medium (2)	226	.77	.83			
	High (3)	16	1.01	1.00			
	Total	390	.83	.83			
Pleading for direct intercession (−)	Low (1)	148	1.00	.77	10.29	.000	1 < 2 1 < 3 2 < 3
	Medium (2)	226	1.26	.75			
	High (3)	16	1.76	.69			
	Total	390	1.18	.77			
Religious focus (+)	Low (1)	148	.87	.77	5.97	.003	1 < 3
	Medium (2)	226	1.05	.79			
	High (3)	16	1.51	.94			
	Total	390	1.00	.80			
Religious purification/forgiving (+)	Low (1)	148	.99	.82	29.89	.000	1 < 2 1 < 3 2 < 3
	Medium (2)	226	1.43	.82			
	High (3)	16	2.48	.77			
	Total	390	1.31	.87			
Spiritual connection (+)	Low (1)	148	.91	.87	22.55	.000	1 < 2 1 < 3 2 < 3
	Medium (2)	226	1.30	.92			
	High (3)	16	2.38	.87			
	Total	390	1.20	.95			
Spiritual discontent (−)	Low (1)	148	1.05	.70	1.06	.346	ns
	Medium (2)	226	1.09	.77			
	High (3)	16	1.33	.72			
	Total	390	1.08	.74			
Marking religious boundaries (+)	Low (1)	148	1.08	.85	6.36	.002	1 < 2 1 < 3
	Medium (2)	226	1.32	.81			
	High (3)	16	1.69	.77			
	Total	390	1.24	.83			
Seeking support from clergy/members (+)	Low (1)	148	.91	.88	5.28	.005	1 < 3 2 < 3
	Medium (2)	226	1.05	.86			
	High (3)	16	1.64	.94			
	Total	390	1.02	.88			
Religious helping (+)	Low (1)	148	.85	.76	17.66	.000	1 < 2 1 < 3 2 < 3
	Medium (2)	226	1.17	.79			
	High (3)	16	1.96	.78			
	Total	390	1.08	.81			
Interpersonal religious discontent (−)	Low (1)	148	1.01	.78	1.42	.242	ns
	Medium (2)	226	1.05	.73			
	High (3)	16	1.34	.68			
	Total	390	1.04	.75			
Religious direction/conversion (+)	Low (1)	148	.94	.82	19.69	.000	1 < 2 1 < 3 2 < 3
	Medium (2)	226	1.27	.87			
	High (3)	16	2.25	.82			
	Total	390	1.19	.89			
Positive religious strategies (+)	Low (1)	148	.98	.68	20.61	.000	1 < 2 1 < 3 2 < 3
	Medium (2)	226	1.27	.68			
	High (3)	16	2.03	.65			
	Total	390	1.19	.72			
Negative religious strategies (−)	Low (1)	148	.99	.68	4.06	.018	1 < 3 2 < 3
	Medium (2)	226	1.06	.67			
	High (3)	16	1.48	.49			
	Total	390	1.05	.67			

Individuals with a high metaphysical sense of quality of life (Group 3) chose some of the positive religious strategies significantly more often than individuals with a low level of this variable (Group 1), namely: *Benevolent Religious Reappraisal/Spiritual Support* (*F* = 2.82, *p* < .001), *Collaborative Religious Coping* (*F* = 11.02, *p* < .001), *Active Religious Surrender* (*F* = 9.49, *p* < .001), *Religious Purification/Forgiveness* (*F* = 29.89, *p* < .001), *Spiritual Connection* (*F* = 22.55, *p* < .001), *Marking Religious Boundaries* (*F* = 6.36, *p* < .01), *Seeking Support From Clergy/Members* (*F* = 5.28, *p* < .01), *Religious Helping* (*F* = 17.66, *p* < .001), *Religious Direction/Conversion* (*F* = 19.69, *p* < .001), and *Positive Religious Coping* treated as a whole (*F* = 2.61, *p* < .001).

Contrary to expectations, individuals with a high metaphysical sense of quality of life (Group 3) chose some negative religious strategies more often than those with a low level of this variable (Group 1), namely: *Punishing God Reappraisal* (*F* = 1.66, *p* < .001), *Demonic Reappraisal* (*F* = 8.50, *p* < .001), *Pleading for Direct Intercession* (*F* = 1.29, *p* < .001), and *Negative Religious Coping* treated as a whole (*F* = 4.06, *p* < .05).

## Discussion

The aim of the study was to analyze differences in religious strategies of coping with stress in a group of prison inmates characterized by different levels of the sense of quality of life—general, psychophysical, psychosocial, personal, and metaphysical.

Of these dimensions of the sense of quality of life, it is the psychophysical aspect of life quality that prison inmates rate the highest—they are satisfied with their health, physical fitness, and external appearance. The lowest scores concern the psychosocial sense of quality of life—the participants feel frustration regarding the need for closeness, belonging, and bond with others, especially their close family, which they are isolated from. The results obtained are consistent with the data available in the literature—imprisonment is accompanied by a deprivation of many needs, and what is particularly acutely experienced is the inadequacy of satisfactory interpersonal relations (Dolińska-Zygmunt and Mokrzyńska 2013; Hołyst 2004; Stepstoe et al. 2006).

Inmates cope with the stress-inducing situation of imprisonment by resorting to religious strategies of coping with stress. They use both positive and negative religious strategies, and the strategy type depends on the level of the sense of quality of life. The present study confirms previous findings concerning the use of religious coping with stress by prison inmates (cf. Allen et al. 2013; Niewiadomska 2011; Pallas 2014).

As expected, individuals with a high sense of quality of life significantly more often choose positive religious strategies compared to the participants with a low sense of quality of life, and also more often compared to those with a medium level of this variable. Those who are generally satisfied with their life more often perceive a difficult situation as potentially advantageous and beneficial—it is an opportunity for them to get closer to God and learn something important (*Benevolent Religious Reappraisal/Spiritual Support*); they also regard religion as helpful in radically changing their life by giving it a new aim and direction (*Religious Direction/Conversion*). The individuals evaluating their general sense of quality of life as high also more frequently cooperate with God in solving their problems (*Collaborative Religious Coping*) and more often feel His closeness (*Spiritual Connection*). The prison inmates who are more satisfied with their life are more willing to engage in religious practices (*Religious Purification/Forgiveness)* and give spiritual support to others (*Religious Helping).* By contrast, those dissatisfied with their life more often choose the strategy of *Passive Religious Deferral*—they shift the burden of solving their problems to God and passively wait for Him to take control of their lives. In the available literature, the opposite relationship has usually been analyzed, the conclusion being that negative religious strategies lower the sense of quality of life—for example, in people with AIDS (Lee et al. 2014) or with advanced cancer (Tarakeshwar et al. 2006).

As regards the psychophysical aspect of the sense of quality of life—as hypothesized, the inmates who rate this dimension of functioning low perceive their situation negatively—as God’s punishment for sins (*Punishing God Reappraisal)* or as a result of Satan’s activity (*Demonic Reappraisal*)—more often than individuals satisfied with their health condition and vitality do. They also more frequently challenge God’s Power (*Reappraisal of God’s Power*) and express their discontent with Him (*Spiritual Discontent),* assuming a similarly critical attitude toward the Church (*Interpersonal Religious Discontent).* On the one hand, they passively wait for God to solve their problems for them (*Passive Religious Deferral),* and on the other hand, they pray for a miracle and for God’s help (*Pleading for Direct Intercession).* Similar results (tough concerning the reverse relationship) were obtained in a group of patients with cancer—individuals who more often used negative religious strategies had a lower sense of quality of life (Pedersen et al. 2013). In a study by Tedrus and colleagues (2013), the choice of negative religious strategies was associated with a lower level of health-related quality of life. An increase in health-related quality of life was observed in patients undergoing kidney dialysis who used positive religious strategies (Ramirez et al. 2012).

The present research confirmed what the literature reports, namely, that the most problematic aspect of imprisonment is the loss of bonds with close others. Low evaluation of the psychosocial sense of quality of life is accompanied, again, by an attitude of passive criticism—both toward God (*Passive Religious Deferral)* and toward the Church (*Interpersonal Religious Discontent).* By contrast, individuals satisfied with interpersonal relationships adopt a more active attitude—they engage in religious practices *(Religious Purification/Forgiveness)* and take up cooperation with God (*Collaborative Religious Coping)* in solving their problems. In the study by Nolan and colleagues (2012), a positive relationship was found between positive religious strategies and the psychosocial sense of quality of life in patients with schizophrenia.

Imprisonment also results in a distorted sense of individuality and autonomy; the more frustrated a person is in this personal aspect of the sense of quality of life, the more likely they are to adopt a passive attitude, expecting someone else—in this case, God—to solve their problems for them and take control of their life (*Passive Religious Deferral*).

Particularly interesting results were obtained regarding the metaphysical sense of quality of life; it turned out that, contrary to expectations, the individuals who evaluate the spiritual sphere high more often choose also certain negative religious strategies besides positive ones, namely: *Punishing God Reappraisal*, *Demonic Reappraisal*, and *Pleading for Direct Intercession.* The last of these strategies is not treated as negative in the Polish population (cf. Talik 2013), and the frequent use of negative strategies can be explained by a generally greater openness to transcendent reality in people with a high metaphysical sense of quality of life, transcendent reality involving the coexistence of the forces of good—God (*Punishing God Reappraisal*) and evil—Satan (*Demonic Reappraisal*). What is puzzling is why individuals to whom religious values are important feel punished by God, which in fact means that they have a negative image of God. The first explanation refers to the universality argument—many people think about God in this way, including many believers, and often without realizing it (Chlewiński 2000; Pargament et al. 2000; Skoblicki 2000; Święs 2006). Secondly, the presence of a caricatural image of God may be explained by negative experiences in relations with significant others, especially with parents, and in the case of prison inmates bad relations in the family are often observed (Gorzelak 2001; Brągiel 1998; Mazur 2005). A caricatural image of God is also a result of personal difficulties (Molenda 2006), which prisoners have. Because religion is an important aspect of functioning for them, they more often choose strategies that make it possible to get closer to God (*Collaborative Religious Coping, Religious Purification/Forgiveness),* whom they regard as close (*Spiritual Connection)*, with whom they actively cooperate (*Collaborative Religious Coping*), and to whom they entrust their problems after attempting to cope with them on their own (*Active Religious Surrender*). They perceive difficult situations as opportunities to get closer to Him (*Benevolent Religious Reappraisal/Spiritual Support*) and consider religion to be helpful in changing their lives (*Religious Direction/Conversion*). Greater satisfaction with the realization of universal values is accompanied by adopting a clear, unambiguous religious attitude (*Marking Religious Boundaries*), in which the community of believers becomes an important source of support (*Seeking Support From Clergy/Members).* Such people are also more willing to give support to others (*Religious Helping*). In the study by Pedersen and colleagues (2013), a positive relationship was observed between the strategy of *Seeking Support From Clergy/Members* and the sense of quality of life in patients with lung disease (Pedersen et al. 2013).

Summing up, as expected, prison inmates more frequently resort to religious strategies in the difficult situation of imprisonment. As predicted, the type of strategies chosen depends on the level of the sense of quality of life. Individuals with a high sense of quality of life—both general and pertaining to specific dimensions—usually choose positive religious strategies, whereas participants with a low sense of quality of life more often choose negative strategies. The exception is the metaphysical aspect of the quality of life: individuals with a high intensity of this dimension more often choose some of the positive as well as negative religious strategies. In other words, the more satisfied a person is with his or her life, the more often he/she employs positive strategies—and conversely, greater dissatisfaction with one’s life is accompanied by more frequent use of negative strategies: by adopting an attitude of passiveness, dissatisfaction, and criticism with regard to God and the Church. The basic mechanism of projection probably operates here—that is, projecting one’s own negative states outside: in this case, on God. Besides, in this way, prison inmates seek the guilty party outside, in addition to passively waiting for their problems to be solved by the Higher Power. Similar results were obtained in the study by Niewiadomska (2007), which showed that prison inmates more often experience a sense of helplessness.

In the studies conducted so far, the impact of religious strategies on the sense of quality of life was analyzed; a positive relationship between positive religious strategies and the quality of life was consistently found (Nolan et al. 2012; Ramirez et al. 2012; Warren et al. 2015). The novelty of the present study is the reverse direction of the analyzed relations—the current project checked how the sense of quality of life influenced the choice of religious strategies of coping with stress: which religious strategies are selected by individuals with different levels of quality of life—general and pertaining to its specific dimensions.

In a majority of the available studies, religious strategies were assessed using the short version of the RCOPE Questionnaire (Brief RCOPE), which provides information on positive and negative religious strategies only. In the presented project, the full version of RCOPE was used, distinguishing specific strategies, both positive (10 strategies) and negative (7 strategies), thanks to which a more precise description of religious coping with stress in a group of prison inmates was obtained.

## Limitations

The main limitation of the present study is its failure to include gender differences in the analyses. Taking into account the results of the studies conducted so far (cf. Pargament 1997), it can be expected that religious strategies of coping with stress used by women will differ significantly from the strategies of this kind used by men, which in turn may be a point of departure for further research.

## Future Research

Another interesting challenge would be to conduct longitudinal research exploring the relations between religious ways of coping with stress and committing crimes. This is all the more important as, firstly, such analyses are lacking, and secondly, there is inconsistency in the findings of the existing studies, which concern not religious strategies directly but broadly understood religious commitment: some of them indicate that religious activity diminishes criminal tendencies among the convicted (O’Connor and Perreyclear 2002; Pallas 2014), while others indicate that religious commitment does not prevent from committing crimes; what is more, sex offenders who described themselves as religiously committed were guilty of more crimes, involving a greater number of younger victims (Eshuys and Smallbone 2006).

Coping with stress is one of the key skills in the process of rehabilitation, which is also not without importance to the safety of the personnel and inmates of the rehabilitation facility (Compas et al. 2001). As the present research has shown, it is important in this process to take religious strategies of coping with stress into account. It seems, though, that the application of these findings—however, important to the rehabilitation of prison inmates—may be difficult in the Polish conditions, given the conditions connected with the bureaucracy in penitentiaries, excessive documentation, and the lack of time on the part of rehabilitation staff for the construction of programs, not to mention their implementation.

## Footnotes

1 The tables below illustrate significant statistical differences only, without showing nonsignificant relationships.
